# Protein and Free Amino Acid Composition of Preterm Human Milk: A Systematic Review and Meta-analysis

**DOI:** 10.1016/j.advnut.2025.100432

**Published:** 2025-04-25

**Authors:** Derek C Miketinas, Mindy A Patterson, Tonya M Bender, Jennifer N Kinnaman, Dixie L Carter, Nathan A Meredith, Katie E Niemeier, Ariana DL Bailey

**Affiliations:** 1 Department of Nutrition and Food Sciences, Texas Woman’s University, Houston, TX, United States; 2 Institute for Women’s Health, Texas Woman’s University, Houston, TX, United States; 3 Global Research & Development, Mead Johnson Nutrition, Evansville, IN, United States

**Keywords:** protein, free amino acids, preterm, human milk, milk, colostrum, transition, mature

## Abstract

The composition of total protein and free amino acids (FAA) in preterm human milk (HM) is crucial for optimizing infant growth and development. The objective of this systematic review and meta-analysis is to quantify the amount of true, crude, and unspecified protein and FAA in preterm HM. EBSCO, PubMed, and Scopus databases were searched up to July 2023 measuring total protein and FAA in preterm HM. Two reviewers, working independently, screened all titles and abstracts using Covidence software to identify studies meeting inclusion criteria [preterm <37 wk; Human Development Index >0.8; cross-sectional, case-controlled (*n >* 1), prospective cohort, and randomized clinical trials; English language]. Random-effects models were used to estimate mean protein and FAA content across studies. Data were aggregated for studies reporting multiple estimates (e.g. across time). Heterogeneity was estimated using *I*^2^ and publication bias using Kendall tau rank correlation coefficient. Of the 884 articles identified, a total of 66 original studies were included for the meta-analysis comprising an estimated 30,421 preterm HM samples. Preterm colostrum (<4 d) contained the highest mean (95% confidence interval) true protein at 2. 32 (1. 96, 2. 68) g/100 mL, followed by transition preterm HM (5–14 d) mean true protein of 1. 77 (1. 60, 1.93) g/100 mL. Mature (>14 d) preterm HM had the lowest mean true protein content at 1.46 (1.34, 1.59) g/100 mL. Glutamate was the most prevalent FAA reported. This systematic review provides updated estimates of protein and FAA concentrations in preterm HM. There was significant variability in the quality of studies, completeness of the reported results, and analytical methodologies across studies.

This trial was registered at PROSPERO as CRD42023445191.

**Statement of significance**

This manuscript advances the field of human milk (HM) nutrition by providing up-to-date estimates of protein and/or free amino acids in colostrum, transition, and mature preterm HM. These values fulfill a critical need for practitioners and researchers to more accurately understand the nutritional needs of preterm infants, as well as any supplementary nutrition that may be necessary for appropriate growth and development.

## Introduction

Early nutrition is critical to support healthy growth and development along with long-term outcomes of infants. Human milk (HM) is the exclusive preferred feeding mode ≤6 mo of age and should continue for ≤2 y of life with additional foods whenever feasible [1,2]. Not only does HM provide vital nutrition for the infant, it also contains a wide range of bioactive components, proteins, and amino acids that promote lean body mass growth, immune function, hormonal regulation, metabolism, and gut integrity [3].

The protein content of HM is known to decline fairly rapidly 2 wk after birth and stabilize to mature levels within ∼ 4–6 wk postpartum [4]. Although this corresponds well to a term infant’s protein requirements, infants born preterm (<37 wk gestation) frequently need much higher enteral protein and amino acids because of a shortened gestational accretion period as well as other factors like fluid intake restrictions and underlying medical conditions [5]. These factors combined make it difficult to meet the nutritional requirements of preterm infants through HM alone. Fortification of preterm HM feeds is therefore required for this population, with protein fortification strategies varying based on body weight and growth goals, which may evolve over the course of hospitalization [6,7]. Consequently, it is important to understand both the protein content and the overall nutrient composition of preterm HM to better tailor fortification and promote optimal growth and development of the preterm infant.

The primary objective of this systematic review and meta-analysis was to quantify the true, crude, and unspecified protein and free amino acid (FAA) content and variability of preterm HM, stratified by postnatal age. Other systematic reviews and meta-analyses of preterm HM nutrient composition have been conducted with some only using studies that included results from 24-h samples [8,9] for all macronutrients although another from 2014 [10] included studies reporting protein content of all samples, regardless of whether they were collected over 24 h or pooled. As protein content of HM is relatively stable over the course of a day with little circadian variation, [11,12] the present study therefore elected to include studies that reported protein measurements regardless of whether they were from pooled samples to capture as many data points as possible. The secondary objective was to stratify total protein by the analytical method. The broader research aim is to conduct a systematic review and meta-analysis of preterm HM nutrient composition and characteristics. The importance of HM for all infants, including those born prematurely, highlights the need for these types of publications.

## Methods

This systematic review and meta-analysis followed the PRISMA 2020 guidelines and was registered at PROSPERO (CRD42023445191; https://www.crd.york.ac.uk/PROSPERO/display_record.php?RecordID=445191). The PRISMA checklist of items in this systematic review and meta-analysis can be found in Supplemental Table 1.

### Search strategy

A research librarian searched EBSCO, PubMed, and Scopus databases through July 2023 to identify intervention and observational studies measuring total protein and/or FAA in preterm HM. The search terms for EBSCO and PubMed included ("Milk, Human/chemistry"[Mesh]) AND ("Premature Birth"[MeSH])) NOT ("systematic review"[Publication Type]) AND “Premature Birth” [MeSH Terms] AND "milk, human"[MeSH Terms] OR "Colostrum"[MeSH Terms]. The search terms for Scopus included TITLE-ABS-KEY ("breast Milk", AND human) AND TITLE-ABS-KEY (preterm OR premature) AND TITLE-ABS-KEY ("Breast Milk" AND analysis) AND (LIMIT-TO (SUBJAREA, "CHEM"). Bioactive nutrients that may confer health benefits to preterm infants and are considered proteins (e.g. lactoferrin, hormones) were not included.

### Eligibility criteria

Inclusion criteria were as follows: studies published until July 2023, studies published in English, and studies that reported HM composition from mothers who delivered preterm defined as gestational age (GA) < 37 wk or identified by authors as preterm. Exclusion criteria included studies whose samples were from countries with a Human Development Index (HDI) <0.8 at time of publication to mitigate risk of maternal malnutrition influencing milk composition. Studies with an *n* = 1 were also excluded.

### Data extraction

Covidence software was used to identify articles meeting inclusion criteria. After duplicates were removed by the software, 2 independent reviewers (DCM and MAP) systematically reviewed the searched titles, abstracts, and full-text articles. Any disagreements were resolved with a third researcher (ADLB).

Two researchers (DCM and MAP) extracted the following data from the included studies: name of first author, publication year, country, study design, sample size of infants, GA, age at which HM was collected, milk type (colostrum, transition, and mature but only if specified), HM collection method, and protein and/or FAA analysis method. Protein composition is estimated from nitrogen content of milk samples. However, nitrogen content consists of protein and nonprotein nitrogen. Therefore, studies reported either true protein (protein nitrogen only), crude protein (total nitrogen), and unspecified protein (could not be determined from manuscript). Any discrepancies in data extraction were reviewed by both reviewers and corrected.

### Statistical analysis

Preterm HM samples were treated as the sampling unit. All analyses were performed by protein type (true protein, crude protein, and unspecified protein). For studies that reported protein and/or FAA across subgroups, the total sample mean and SD were estimated. Supplemental Table 2 provides the data handling steps for how missing data were treated. For randomized controlled trials (RCTs), baseline or control group milk composition estimates were extracted. For studies that compared analytical methods, only the reference analytical method (i.e. Kjeldahl) was included.

This meta-analysis was conducted in R using the metafor package [13] to fit random-effects models to estimate protein and FAA content across HM type [colostrum (≤ 4 d), transition (5–14 d), and mature (>14 d)]. The protein of mature HM was further stratified by 14–42 d, 43–84 d, and >84 d [4]. For studies that reported protein content, subgroup analyses were conducted across analytical method: Kjeldahl, bicinchoninic acid (BCA)/Lowry, infrared (IR) spectroscopy (near- or mid-), and other/not specified. Estimates were aggregated for studies that reported multiple collections per milk type. Heterogeneity was tested using Cochrane-*I*^2^ statistics. The level of heterogeneity (*I*^2^) was measured as a percentage where <40% is low, 40%–75% is moderate, and ≥75% is high heterogeneity. Publication bias was assessed using Kendall’s tau rank correlation coefficient. Sensitivity analysis was conducted to evaluate the impact of each study on the pooled estimates.

## Results

### Study selection

**FIGURE 1:**
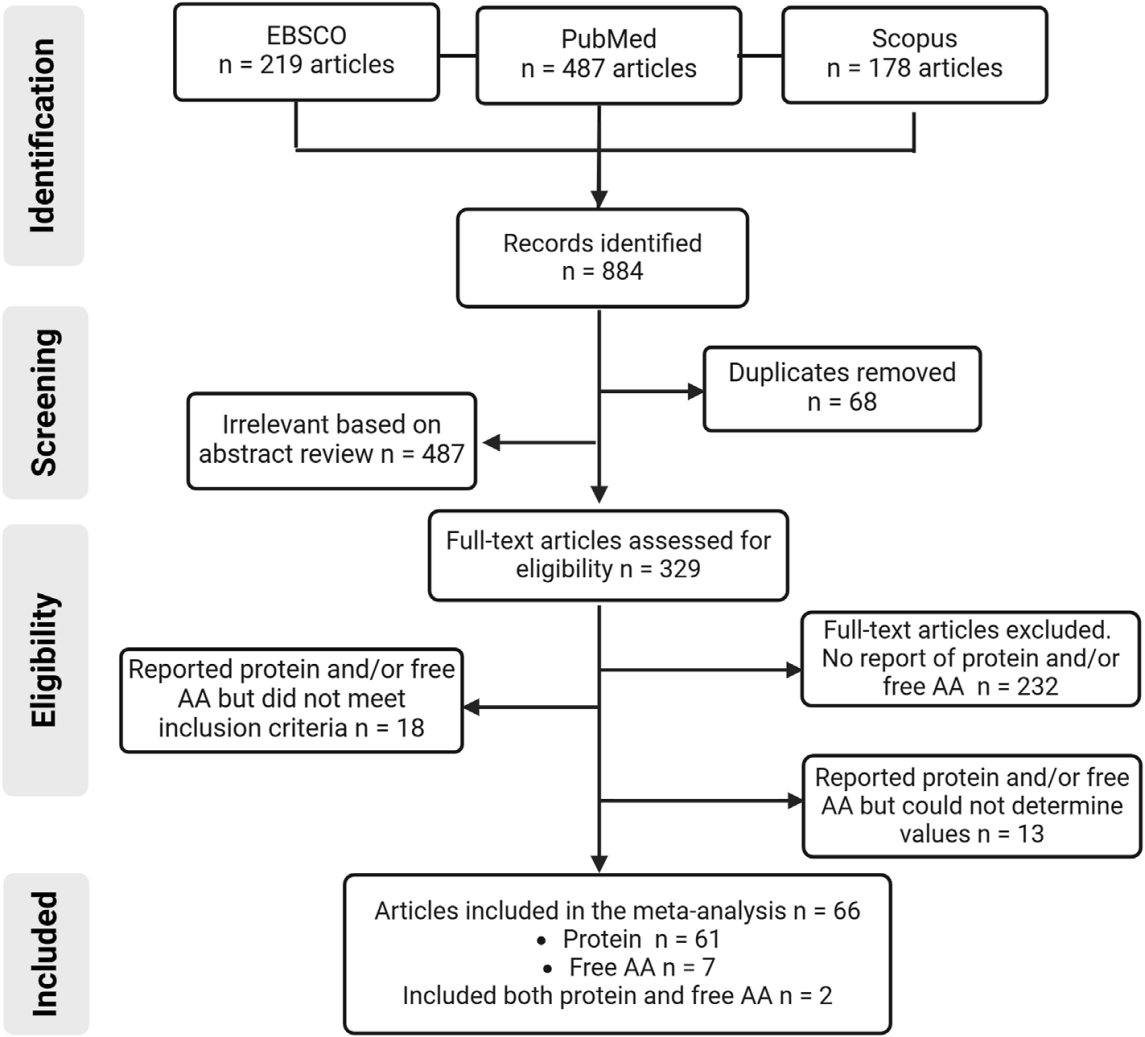
Study selection flowchart. AA, amino acids.

Of the 884 studies identified, 68 were removed as duplicates, 487 were considered irrelevant based on title and abstract review leaving 329 studies that involved full-text review (Figure 1). A total of 97 studies reported protein or FAA preterm HM values. An additional 31 studies reporting protein and/or FAA were further excluded (18 did not meet inclusion criteria; 8 provided results in a figure without values; 5 did not measure or provide protein values). Thus, 66 articles were included in the analysis (61 protein, 7 FAA, and 2 of which reported both) comprising an estimated 30,421 preterm HM samples for protein and 251 for FAA.

### Study results

Table 1 [[14], [73]] describes study characteristics for preterm HM protein included in the analyses. Thirty-one reported true protein content, 10 reported crude protein, 2 reported total and true protein, and 18 did not specify. These studies included data from mothers of 2, 669 infants. Thirty-seven of the studies employed a longitudinal design, 16 were cross-sectional, and 8 were RCTs. Twenty-one (34%) were conducted in the United States and 22 (36%) were conducted in Europe. The most common method for analyzing protein content among the studies included was IR spectroscopy followed by the Kjeldahl method.

**TABLE 1:** Summary of the characteristics of studies reporting protein (*n* = 61)^1^

Author	Year	Country	Study design	Sample size infants	Gestational age^1^ (wk)	Breastmilk age^2^ (d)	Milk type^3^	HM collection method	Nutrient	Protein analysis method
Abdulrazzaq, Y [15]	2003	UAE	CS	49	<37.0	1	C	Pump, electric	Protein unspecified	Milkoscan FT120
Anderson, D [16]	1983	United States	L	14	31	(3, 14)	C, M, T		True protein	Kjeldahl
Anderssen, S [17]	2015	Norway	L	47	31	(13, 30)	T, M	Pump, electric	Protein unspecified	Mid-infrared spectroscopy
Bauer, J [18]	2011	Germany	L	102	28	(7, 56)	T, M	Pump, electric	True protein	BCA/Lowry
Beijers, R [19]	1992	Netherlands	L	45	30.7	(3, 14)	C, M, T	Pump, electric	True protein	Other
Belfort, M [20]	2020	United States	L	37	28	58.5	M	Pump, electric	True protein	Mid-infrared spectroscopy
Bishara, R [21]	2008	United States	CS	30	(24.0, 27.9)	24	M	Pump, electric	Crude protein	Kjeldahl
Brion, L [22]	2020	United States	RCT	58	28				Protein unspecified	Near-infrared spectroscopy
Britton, J [23]	1986	United States	CS	70	(25.0, 35.0)	(3, 25)	C, M, T	Pump, electric	True protein	BCA/Lowry
Bulut, Ö [24]	2019	Turkey	L	32	(25.0, 33.0)	(7, 42)	C, M, T	Pump, electric	True protein	Mid-infrared spectroscopy
Butte, N [25]	1984	United States	L	8	33.9	(14, 84)	T	Pump, electric	True protein	Kjeldahl
Caldeo, V [26]	2021	Ireland	L	39	33.7	38.5	M	Manual and pump, electric	True protein	BCA/Lowry
Campbell-Yeo, M [27]	2010	Canada	RCT	24	26.8	(0, 14)	C, T	Pump, electric	Crude protein	Kjeldahl
Chuang C [28]	2005	Taiwan	CS	23	33	10.5	T		True protein	BCA/Lowry
Corvaglia, L [29]	2008	Italy	CS	55	(26.0, 32.0)	10	T		Crude protein	Mid-infrared spectroscopy
Corvaglia, L [30]	2010	Italy	CS	34	(24.0, 33.0)	22	M		True protein	Near-infrared spectroscopy
Dingess, K [31]	2017	Netherlands	CS	12	29.1	73	M	Pump, unspecified	True protein	BCA/Lowry
de Halleux, V [32]	2013	Belgium	L	28	28.6	28	M	Manual and pump, electric	Protein unspecified	Milkoscan FT120
de Oliveira, S [33]	2017	France	RCT	12	30	27	M		Protein unspecified	Other
Ellis, L [34]	1990	United States	L	22	31	(3, 42)	C, M, T	Pump, electric	True protein	BCA/Lowry
Elmlinger, M [35]	2007	Germany	L	30	(24.0, 31.0)	(7, 21)	T, M	Pump, electric	True protein	BCA/Lowry
Erickson, T [36]	2013	United States	L	8	-4	14	T		True protein	BCA/Lowry
Faerk, J [37]	2001	Denmark	L	101	28	(11, 67)	T, M	Pump, electric	True protein	Milkoscan 104
Gates, A [38]	2021	United States	L	38	28.2	(7, 28)	T, M		Crude protein	Combustion
Groh-Wargo, S [39]	2016	United States	CS	10	(23.9, 32.4)	25	M	Pump, electric	True protein	Kjeldahl
Gross, S [1]	1980	United States	L	33	31.4	(3, 28)	C, M, T	Manual and pump, electric	Crude protein	Kjeldahl
Gross, S [40]	1983	United States	RCT	20	(27.0, 33.0)	(7, 70)	T, M		Crude protein	Kjeldahl
Gross, S [41]	1987	United States	RCT	19	<30.2	(7, 35)	T, M		Crude protein	Kjeldahl
Hsu, Y [42]	2014	Taiwan	L	17	29	(6, 27)	T, M	Manual and pump, unspecified	Protein unspecified	Mid-infrared spectroscopy
Kreissl, A [43]	2016	Austria	L	76	<32.0	(7, 28)	T, M	Pump, electric	True protein	Mid-infrared spectroscopy
Lemons, J [44]	1982	United States	L	20	33	(7, 56)	T, M	Pump, electric	Crude protein, true protein	Kjeldahl
Lemons, J [45]	1983	United States	L	20	33	25.5	M	Pump, electric	Crude protein, true protein	Kjeldahl
Lepage, G [46]	1984	Canada	CS	32	(26.0, 36.0)	30	M	Manual and pump, electric	Crude protein	Kjeldahl
Lev, H [47]	2014	Israel	L	20	30.6	31.5	M	Pump, unspecified	Protein unspecified	Mid-infrared spectroscopy
Maas, C [48]	2017	Germany	RCT	60	<32.0	(14, 28)	T, M		Protein unspecified	Mid-infrared spectroscopy
Maas, Y [49]	1998	Netherlands	L	79	<30.0	(9, 53)	T, M	Manual and pump, electric	Crude protein	Kjeldahl
Maly, J [50]	2019	Czech Rep	L	225	(24.0, 35.0)	(7, 63)	T, M	Manual and pump, electric	True protein	Mid-infrared spectroscopy
McLeod, G [51]	2013	Australia	L	63	30	14	T		True protein	Kjeldahl
McLeod, G [52]	2015	Australia	L	27	29	40	M		True protein	Kjeldahl
Minić, S [53]	2018	Serbia	CS	20	(28.0, 36.0)	12	T		Protein unspecified	Not specified
Molinari, C [54]	2013	Australia	L	17	30.7	(7, 14)	T	Pump, electric	True protein	Other
Montagne, P [55]	1999	France	CS	46	<37.0	(3, 12)	C, T		True protein	Other
Moran-Lev, H [56]	2015	Israel	L	32	30.1	28	M	Pump, electric	Protein unspecified	Mid-infrared spectroscopy
Morton, J [57]	2012	United States	L	52	<31.0	(7, 56)	T, M	Manual and pump, electric	True protein	Other
Nielsen, S [58]	2020	United States	CS	9	26.8	24.5	M	Pump, electric	True protein	BCA/Lowry
Norrgrann, M [59]	2023	Sweden	CS	12	28.1	9.5	T	Pump, electric	Protein unspecified	Mid-infrared spectroscopy
Paulaviciene, I [60]	2020	Lithuania	CS	27	30.2	15	M	Manual and pump, electric	Protein unspecified	Mid-infrared spectroscopy
Perrella, S [61]	2015	Australia	RCT	23	(28.0, 34.0)				True protein	Other
Radmacher, P [62]	2013	United States	CS	83	<1500 g BW^4^	(7, 35)	C, M, T		Protein unspecified	Mid-infrared spectroscopy
Saarela, T [63]	2005	Finland	L	36	31.4	(7, 180)	T, M	Manual and pump, electric	Protein unspecified	Kjeldahl
Sahin, S [64]	2020	Turkey	L	39	29.7	(3, 28)	C, M, T	Manual and pump, hand	Protein unspecified	Mid-infrared spectroscopy
Sann, L [65]	1981	France	L	41	(26.0, 35.0)	(6, 15)	T, M	Manual and Pump, electric	True protein	Other
Sauer, C [66]	2017	United States	L	18	<37.0	31	M	Pump, electric	True protein	Kjeldahl
Smilowitz, J [67]	2014	United States	L	5	<37.0	185	M		True protein	Kjeldahl
Stein, H [68]	1986	South Africa	RCT	11	33.5	(5, 33)	T, M		Protein unspecified	Not Specified
Stevens, L [69]	1969	Australia	L	10	34.8	(5, 27)	T, M	Manual and pump, unspecified	True protein	Kjeldahl
Stoltz Sjöström, E [70]	2014	Sweden	L	256	25.3	(6, 106)	T, M	Pump, electric	True protein	Mid-infrared spectroscopy
Tanaka, M [71]	2023	Japan	CS	26	29	70	M	Manual and pump, unspecified	Protein unspecified	Mid-infrared spectroscopy
Thomas, M [72]	1986	United States	L	8	(30.0, 34.0)	16	T, M	Manual	True protein	BCA/Lowry
Trend, S [14]	2016	Australia	L	45	30.2	(4, 28)	C, M, T	Pump, electric and hand	Crude protein	BCA/Lowry
Zachariassen, G [73]	2013	Denmark	L	214	<32.0	(14, 84)	T, M	Pump, unspecified	Protein unspecified	Mid-infrared spectroscopy

Abbreviations: BCA, bicinchoninic acid; C, colostrum; CS, cross-sectional; GA, gestational age; HM, human milk; IR, infrared; L, longitudinal; M, mature; RCT, randomized controlled trial; RL, randomized longitudinal; T, transition.

1 Values reported as mean or (range)

2 Mean gestational age (GA). Parentheses indicate upper and lower ranges.

3 Mean age human milk (HM) was collected. Parentheses indicate upper and lower ranges.

4 Listed only if article specified.

#### True protein

**FIGURE 2:**
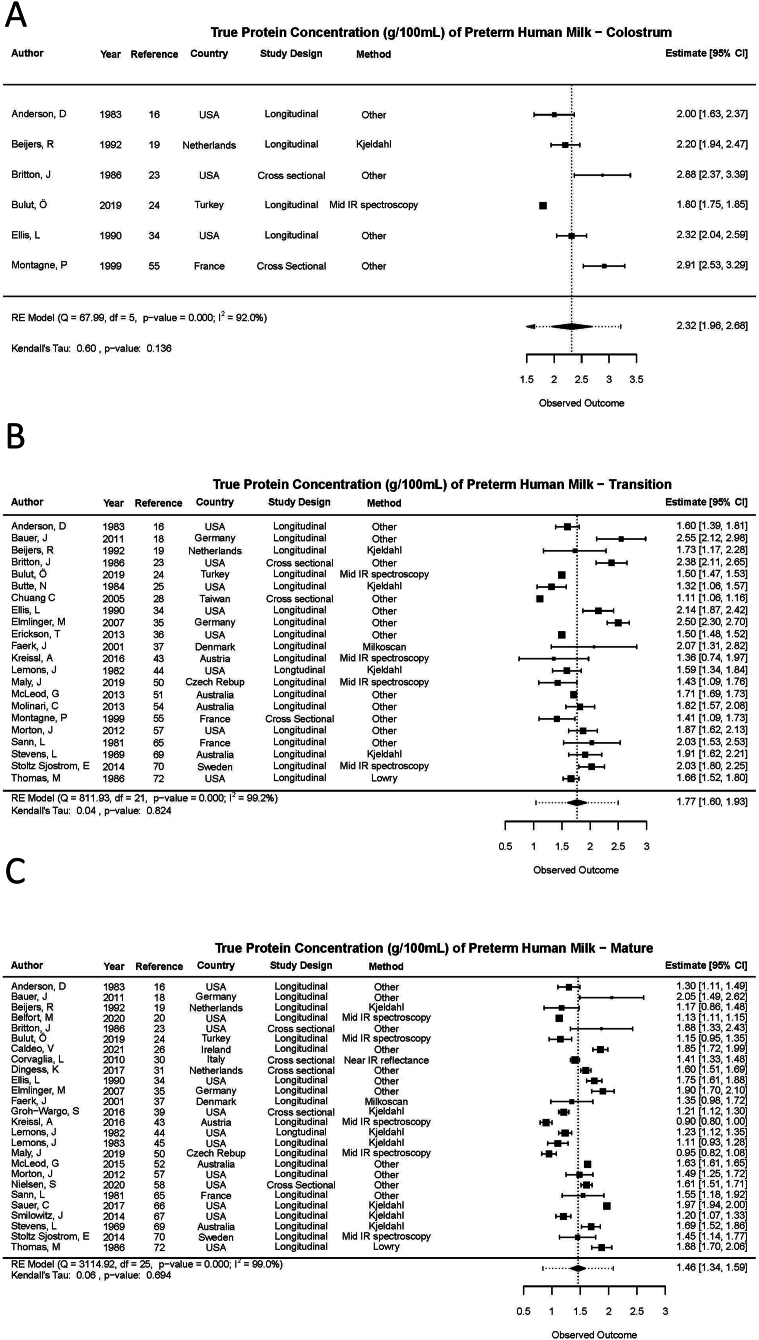
True protein content (g/100 mL) in preterm human milk (HM) across milk type. (A) Colostrum HM (<4 d), RE model (*Q* = 68, df = 5, *P* value <0.001; *I*^2^ = 92.0%); Kendall’s tau: 0.60, *P* value = 0.136. (B) Transition HM (5–14 d), RE model (*Q* = 812, df = 21, *P* value <0.001, *I*^2^ = 99.2%); Kendall’s tau: 0.04, *P* value = 0. 824. (C) Mature HM (>14 d), RE model (*Q* = 3115, df = 25, *P* value <0.001, *I*^2^ = 99.0%); Kendall’s tau: 0.06, *P* value = 0. 694. CI, confidence interval.

**FIGURE 3:**
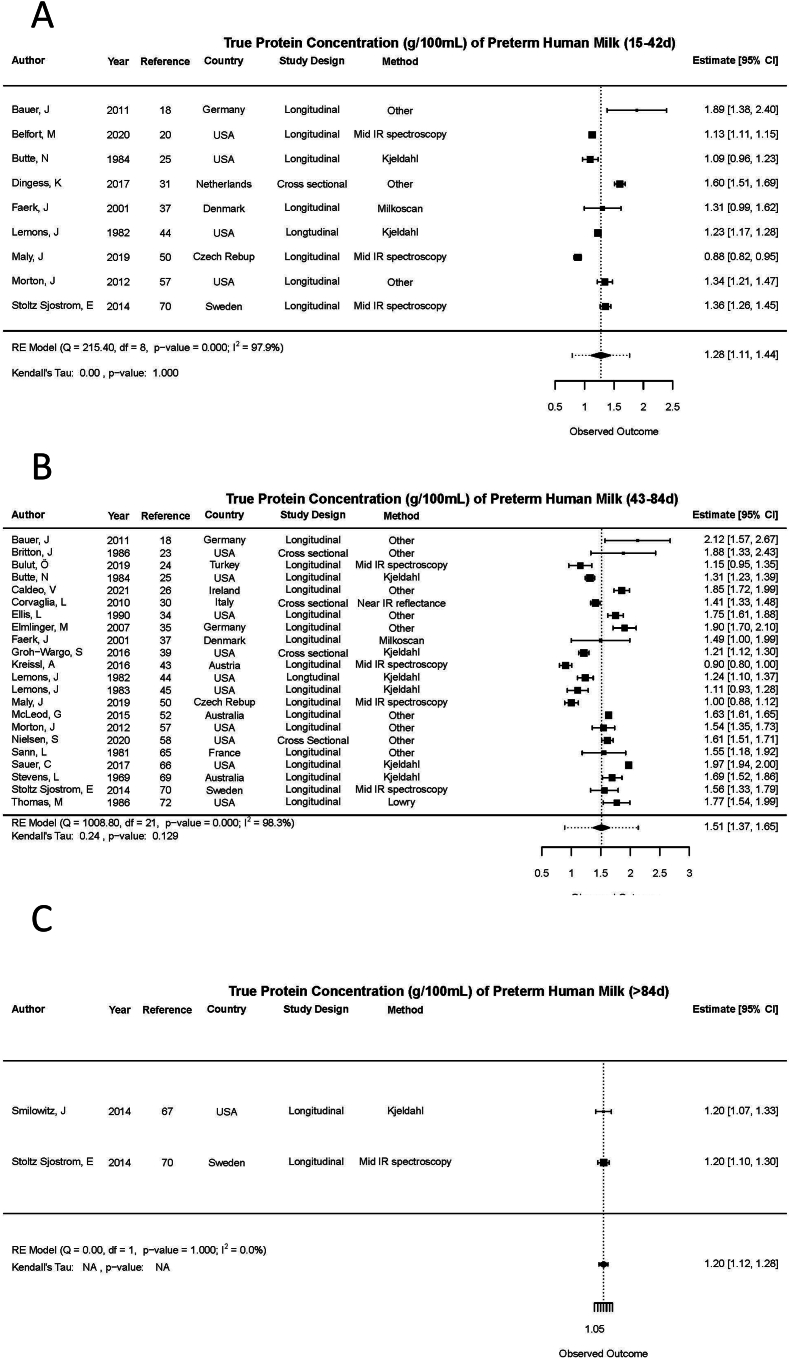
True protein content (g/100 mL) in mature preterm human milk (HM) across time. (A) Mature HM (14–42 d), RE model (*Q* = 1008.8, df = 21, *P* value < 0.001; *I*^2^ = 98.3%); Kendall’s tau: 0.24, *P* value = 0.129. (B) Mature HM (43–84 d), RE model (*Q* = 215.40, df = 8, *P* value < 0.001, *I*^2^ = 97.9%); Kendall’s tau: 0.00, *P* value = 1.000. (C) Mature HM (>84 d), RE model (*Q* = 0.00, df = 1, *P* value = 1.000, *I*^2^ = 0.0%); Kendall’s tau: NA, *P* value = NA.

True protein content in each preterm milk type is provided in Table 2, and the forest plots are provided in Figure 2. Mean [95% confidence interval (CI)] protein content across studies that analyzed colostrum was 2. 32 (1. 96, 2. 68) g/100 mL. Across studies that analyzed transition HM and mature HM, protein content was 1.77 (1. 60, 1.93) and 1.46 (1.34, 1.59) g/100 mL, respectively. Overall, there was high heterogeneity among studies (*I*^2^ ≥ 92.0%) and no evidence of publication bias (*P* > 0.05). Moreover, sensitivity analyses did not reveal any significant departure from the study effect size (Supplemental Figures 1–9). Figure 3 displays true protein content within mature preterm HM and stratified across time-periods postpartum. True protein content of mature HM decreased as postpartum period increased from 1.51 (1.37, 1.65) g/100 mL at 14–42 d, 1.28 (1.11, 1.44) g/100 mL at 43–84 d, and 1.20 (1.12, 1.28) g/100 mL at >84 d.

**TABLE 2:** Meta-analysis results of true, crude, and unspecified protein composition in preterm human milk stratified by milk type

Protein type	Milk type^1^	Estimate^2^	95% Confidence intervals		*Q*	df	*P* value	*I*^2^	Kendall's tau	*P* value
			Lower^2^	Upper^2^						
True protein										
	Colostrum	2.32	1.96	2.68	68	5	< 0.001	92.0	0.60	0.136
	Transition	1.77	1.60	1.93	812	21	< 0.001	99.2	0.04	0.824
	Mature	1.46	1.34	1.59	3115	25	< 0.001	99.0	0.06	0.694
Crude protein										
	Colostrum	2.43	1.54	3.32	35	2	< 0.001	94.7	1.00	0.333
	Transition	1.92	1.72	2.11	67	8	< 0.001	85.2	−0.17	0.612
	Mature	1.55	1.40	1.69	119	11	< 0.001	88.8	−0.09	0.737
Unspecified protein										
	Colostrum	1.88	1.66	2.09	35	2	< 0.001	93.8	−1.00	0.333
	Transition	1.83	1.65	2.01	204	9	< 0.001	97.1	0.24	0.381
	Mature	1.35	1.20	1.49	666	13	< 0.001	98.2	0.21	0.331

1 Colostrum, transition, and mature milks were considered as milk collected < 5 d, 5–14 d, and > 14 d postpartum, respectively.

2 Values are presented in g/100 mL.

#### Crude protein

Crude protein estimates were slightly greater than true protein estimates (Table 2).

Mean (95% CI) crude protein content across studies that analyzed colostrum was 2.43 (1.54, 3.32) g/100 mL. Across studies that analyzed transition HM and mature HM, crude protein content was 1.92 (1.72, 2.11) and 1.55 (1.40, 1.69) g/100 mL, respectively. Overall, there was high heterogeneity among studies (*I*^2^ ≥ 85.2%) and no evidence of publication bias (*P* > 0.05). Sensitivity analyses indicated a potential influential study [14] (Trend 2016) that, if removed, slightly increased the point estimate and narrowed the 95% CI for transition milk to 1.97 (1.91, 2.02) g/100 mL (Supplemental Figures 1–9).

#### Unspecified protein

Studies that did not distinguish the nitrogen source were analyzed together as unspecified protein. These estimates were comparable with true protein estimates (Table 2). Mean (95% CI) unspecified protein content across studies that analyzed colostrum was 1.88 (1.66, 2.09) g/100 mL. Across studies that analyzed transition HM and mature HM, unspecified protein content was 1.83 (1.65, 2.01) and 1.35 (1.20, 1.49) g/100 mL, respectively. Overall, there was high heterogeneity among studies (*I*^2^ ≥ 93.8%) and no evidence of publication bias (*P* > 0.05). Moreover, sensitivity analyses did not reveal any significant departure from the study effect size (Supplemental Figures 1–9).

#### Protein content by methodology

Table 3 displays the differences in true, crude, and unspecified protein content of mature preterm HM by methodology. BCA/Lowry combined with “other” (e.g. combustion) methodology resulted in the highest protein content estimate for true (1.68 g/100 mL) and unspecified (2.10 g/100 mL) protein. The Kjeldahl method reported the second highest estimates for true and unspecified protein (1.46 g/100 mL and 1.50 g/100 mL, respectively) followed by IR spectroscopy (1.26 g/100 mL and 1.48 g/100 mL, respectively). For crude protein content, only the Kjeldahl methodology was reported (1.68 g/100 mL).

**TABLE 3:** Meta-analysis results of true, crude, and unspecified protein composition in preterm human milk stratified by analytical methods

	Method^1^	Estimate^2^	95% Confidence interval		*Q*	df	*P* value	*I*^2^	Kendall's tau	*P* value
			Lower^2^	Upper^2^						
True protein										
	Kjeldahl	1.46	1.23	1.68	441	8	< 0.001	95.8	0.28	0.358
	Infrared	1.26	1.11	1.41	48	6	< 0.001	80.2	0.14	0.773
	Other	1.68	1.55	1.82	671	16	< 0.001	98.9	−0.06	0.776
Crude protein										
	Kjeldahl	1.68	1.50	1.87	107	10	< 0.001	89.6	0.09	0.761
Unspecified protein										
	Kjeldahl	1.50	1.27	1.74	1	1	0.335	0.0	−1.00	1.000
	Infrared	1.48	1.30	1.65	642	12	< 0.001	98.0	−0.03	0.952
	Other	2.10	1.78	2.42	6	2	0.046	72.1	−1.00	0.333

Estimates do not distinguish milk type (i.e. colostrum, transition, mature).

Abbreviation: BCA, bicinchoninic acid.

1 Other includes BCA/Lowry, other, and nonspecified methods.

2 Estimates are presented in g/100 mL.

#### Free amino acids

**FIGURE 4:**
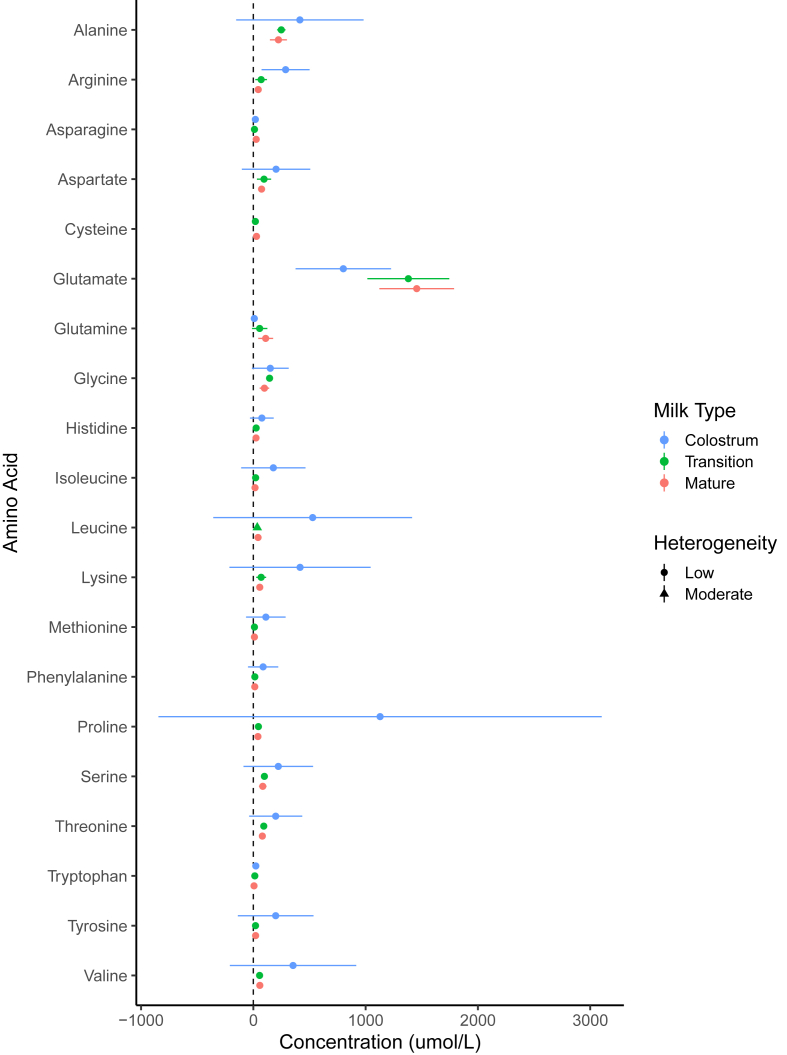
Forest plots for free amino acid concentrations (μmol/L) across milk type. Milk type was denoted by color: colostrum (), transition (), and mature (). Low and moderate heterogeneity was defined as <40% and 40%–75%, respectively.

The characteristics of studies reporting FAA can be found in Table 4 [28,45,46,[74], [75], [76], [77]]. The FAA meta-analysis results can be found in Figure 4 and the numerical estimates can be found in Supplemental Table 3. Proline (1129 μmol/L) and glutamate (801 μmol/L) were the most predominant FAA found in preterm colostrum. Interestingly, glutamate increased to 1380 and 1455 μmol/L in transition and mature preterm HM, respectively, and was the most predominant FAA in those phases. Glutamine also increased in preterm HM across the 3 phases. Glutamine was lowest in colostrum (8.5 μmol/L) followed by transition (56.1 μmol/L), then mature (109.7 μmol/L). Alanine was the second most prevalent FAA in transition (249 μmol/L) and mature (223 μmol/L) preterm HM.

**TABLE 4:** Summary of the characteristics of studies reporting free amino acids in preterm HM (*n* = 7)^1^

Author, first	Year	Country	Study design	Infants, *n*	GA^2^ (wk)	HM age^3^ (d)	HM type^4^	HM collection method	FAA analysis method
Chuang, C [28]	2005	Taiwan	CS	23	33	(4, 21)	C, T, M		IE chromatography with UV–vis detection
De Oliveria, S [74]	2016	France	CS	5	29.8	46.2	M		IE chromatography with UV–vis detection
Lemons, J [45]	1983	United States	L	20	33	31.5	M	Pump, electric	IE chromatography with UV–vis detection
Lepage, G [46]	1984	Canada	CS	32	(26.0, 36.0)	(8, 21)	T, M	Manual and pump, electric	IE chromatography with UV–vis detection
Pamblanco, M [75]	1989	Spain	CS	26	(26.0, 36.0)	(3, 14)	C, M	Pump, electric	RP-HPLC with fluorescence detection
RiveraVelez, S [76]	2023	United States	CS		28.6	(3, 14)	C, T, M		LC-MS/MS
Spevacek, A [77]	2015	United States	L	13	29.8	(3, 28)	C, T, M	Pump, hand	NMR

Abbreviations: C, colostrum; CS, cross-sectional; FAA, free amino acids; IE, ion-exchange chromatography; LC-MS/MS, liquid chromatography tandem mass spectrometry; L, longitudinal; M, mature; NMR, nuclear magnetic resonance; RP-HPLC, reverse phase high-performance liquid chromatography; T, transition.

1 Values reported as mean or (range).

2 Mean gestational age (GA). Parentheses indicate upper and lower ranges.

3 Mean age human milk (HM) was collected. Parentheses indicate upper and lower ranges.

4 Listed only if article specified.

## Discussion

Characterizing protein and FAA composition of preterm HM over the course of lactation is foundational to understanding this population’s unique needs. The mean protein contents reported here across colostrum, transition, and mature preterm HM are in agreement with previous systematic reviews [[7], [8], [9], [10]]. Although protein is considered the key driver of lean body mass growth, the amino acid composition of HM is important to characterize as both protein and amino acids are critical for neurodevelopment. Indeed, inadequate amino acid supply to the preterm infant has been shown to result in suboptimal brain development and correspondingly reduced neurodevelopment outcomes later in life [78,79]. Although this study only examined FAA and not total amino acids composition, characterizing the amino acid composition of preterm HM is necessary as that is what ultimately serves as the baseline for HM substitutes. Future studies and reviews should aim to assess total amino acid composition as well as that is the most accurate way of estimating true protein content [80] and the majority of amino acids in HM are found as intact proteins or peptides rather than FAA.

Of the FAA in HM, glutamate and glutamine content of preterm HM consistently increased over the course of lactation. The increase of these specific FAA over time suggests they may play an important role in the developing preterm infant. This agrees with a 2014 systematic review of amino acid composition of preterm and term HM, which found glutamine in mature preterm HM to be 20 times higher than in colostrum [81]. Glutamine and glutamate are reported to support intestinal function by increasing the growth of intestinal epithelial cells and supporting the intestinal barrier [82]. Receptors for these FAA are also found on various immune cells, suggesting an immunomodulatory role of glutamine and glutamate. Contrastingly, the remaining FAA reported here decreased over the course of lactation, with some declining sharply after the colostrum phase. This aligns with the trends seen in protein, which also decreases over the course of lactation and highlights the gap with expert bodies’ recommendations for increased protein needs of the preterm infant and parenteral essential amino acid supplementation for neonates born <32 wk gestation. Establishing clinical evidence in infants for the physiological importance of why some FAA are preferentially increased whereas others decrease drastically over the course of lactation may be an important next step for HM research.

Our analysis comparing the protein content of mature preterm HM by methodology revealed that the BCA/Lowry method resulted in the highest protein content estimate; however, it is worth noting that the use of this methodology was lower than the more commonly employed Kjeldahl or IR spectroscopy methods. Infrared spectroscopy providing the lowest protein content estimates agrees with previous literature that indicates this method has the tendency to underestimate protein content compared with other methods like Kjeldahl [83], which has been described to be the most accurate indirect determination of protein content in HM [84,85] and thus is considered to be the method of reference for protein measurement.

The studies included in this systematic review and meta-analysis, and consequently the present analysis, are not without limitations. Of the studies that reported a collection type, most indicated a 24-h or pooled sample for analysis; however, many of the studies included in this review did not mention whether the milk type analyzed was foremilk, hindmilk, whole breast, or pooled. For protein content, this is less of a concern as it remains fairly stable over a 24-h period [11,12]. The sample collection protocol should still be considered for nutrients that exhibit circadian variations or fore/hind milk variations. GA was also frequently unreported or reported without a corresponding range or distribution. This could impact the overall nutrient composition of the milk sample, which presents another limitation. Additionally, a majority did not report the time-of-day milk was collected or how the milk was transferred and stored. Those that reported storage typically indicated that samples were either refrigerated or frozen. Understanding how storage, length of storage, and transfer of milk to storage (e.g. how long a sample was left at room temperature, time it took to transfer to a fridge/freezer, freezing then thawing) may be important future considerations when standardizing an approach to HM research. Some studies also failed to report ideal summary statistics for protein concentration. For example, some studies reported minimum and maximum values instead of SD or reported only means and no variability estimates. The studies included in this review generally lacked maternal descriptives such as BMI, smoking status, parity, and age, [86] all of which may impact protein content. Furthermore, this analysis only used studies that provided protein estimates across countries with an HDI >0.8, so this analysis may not be representative of the nutritional composition of HM from women in developing countries. In addition, some [87] but not all [88] studies have suggested that pasteurization may impact protein and FAA quality and quantity, which should be considered when estimating these values in donor milk, which often comes from late-lactation term donors. Thus, it should be advised that the results of the present systematic review and meta-analysis do not apply to donor milk composition as those values may differ dramatically [89]. Finally, an accurate quality assessment tool could not be identified to grade the quality of each reference as poor, fair, or good. The National Heart, Lung, and Blood Institute’s Quality Assessment Tool for Observational Cohort and Cross-sectional Studies included several assessment questions on exposure; however, most referenced studies in the present analysis did not include an exposure.

This systematic review and meta-analysis of preterm HM provides the most recent estimates of true protein, crude protein, and FAA content across lactation stages. The most recent preterm infant intake recommendation for protein is 3.2–4.1 g of protein per 100 kcal (or 3.5–4.5 g/kg/d), which equates to ∼2.1–2.7 g/100 mL [5]. Our results showed that protein concentration in preterm colostrum may be sufficient for meeting these guidelines; however, the protein concentrations of transition and mature fall well below this range. Furthermore, when following standard feeding volumes of 160–180 mL/kg/d, the protein intake target would not be met unless fed at an impractically high volume, and this is further complicated when considering some preterm infants’ fluid restrictions. Thus, the data presented here not only support clinicians in navigating fortification strategies but also provide industry researchers with an appropriate benchmark to use when innovating fortifiers intended for preterm infants.

## Author contributions

The authors’ responsibilities were as follows – DCM, ADLB, TMB: designed the research (project conception, development of overall research plan, and study oversight); DCM, MAP: conducted the research (hands-on conduct of the experiments and data collection) and provided essential databases necessary for the research; DCM: analyzed data or performed statistical analysis, primary responsibility for final content; DCM, MAP, ADLB: wrote paper (only authors who made a major contribution); DLC, NAM: provided analytical methodology expertise; DLC, NAM, JNK, TMB, KEN, ADLB: provided technical expertise; and all authors: read and approved the final manuscript.

## Data availability

Data described in the manuscript, code book, and analytic code will be made available upon request pending application and approval.

## Funding

Project partially funded by Mead Johnson Nutrition. ADLB, TMB, DLC, JNK, and NAM are employees of Mead Johnson Nutrition. DCM and MAP received funding from Mead Johnson Nutrition to perform the methods and results. The employees of Mead Johnson Nutrition provided input on the study objectives, interpretation of results, and final manuscript, but they did not provide input on the data extraction or statistical analysis.

## Conflict of interest

DCM reports that financial support and writing assistance were provided by Mead Johnson Nutrition. TMB, JNK, DLC, NAM, KEN and ADLB reports a relationship with Mead Johnson Nutrition Company that includes: employment. DCM reports a relationship with Mead Johnson Nutrition Company that includes: funding grants. Consultant for Reckitt | Mead Johnson as an independent contractor on a separate project – DCM. All other authors report no conflicts of interest.

## References

[1] Gross S.J., David R.J., Bauman L., Tomarelli R.M. (1980). Nutritional composition of milk produced by mothers delivering preterm. J. Pediatr..

[2] Meek J.Y., Noble L. (2022). Policy statement: breastfeeding and the use of human milk. Pediatrics.

[3] Dror D.K., Allen L.H. (2018). Overview of nutrients in human milk. Adv. Nutr..

[4] Ballard O., Morrow A.L. (2013). Human milk composition: nutrients and bioactive factors. Pediatr. Clin. North Am..

[5] Koletzko B., Wieczorek S., Cheah F., Domellöf M., van Goudoever J., Poindexter B., Koletzko B., Cheah F., Domellöf M., Poindexter B., Vain N., van Goudoever J. (2021). Nutritional Care of Preterm Infants: Scientific Basis and Practical Guidelines.

[6] Arslanoglu S., Boquien C.Y., King C., Lamireau D., Tonetto P., Barnett D. (2019). Fortification of human milk for preterm infants: update and recommendations of the European Milk Bank Association (EMBA) working group on human milk fortification. Front. Pediatr..

[7] Brown J.V., Lin L., Embleton N.D., Harding J.E., McGuire W. (2020). Multi-nutrient fortification of human milk for preterm infants. Cochrane Database Syst. Rev..

[8] Boyce C., Watson M., Lazidis G., Reeve S., Dods K., Simmer K. (2016). Preterm human milk composition: a systematic literature review. Br. J. Nutr..

[9] Mimouni F.B., Lubetzky R., Yochpaz S., Mandel D. (2017). Preterm human milk macronutrient and energy composition: a systematic review and meta-analysis. Clin. Perinatol..

[10] Gidrewicz D.A., Fenton T.R. (2014). A systematic review and meta-analysis of the nutrient content of preterm and term breast milk. BMC Pediatr.

[11] Leghi G.E., Middleton P.F., Netting M.J., Wlodek M.E., Geddes D.T., Muhlhausler B.S. (2020). A systematic review of collection and analysis of human milk for macronutrient composition. J. Nutr..

[12] Italianer M.F., Naninck E.F.G., Roelants J.A., van der Horst G.T.J., Reiss I.K.M., Goudoever J.B.V. (2020). Circadian variation in human milk composition, a systematic review. Nutrients.

[13] Viechtbauer W. (2010). Conducting meta-analyses in R with the metafor package. J. Stat. Softw..

[14] Trend S., Strunk T., Lloyd M.L., Kok C.H., Metcalfe J., Geddes D.T. (2016). Levels of innate immune factors in preterm and term mothers' breast milk during the 1st month postpartum. Br. J. Nutr..

[15] Abdulrazzaq Y.M., Osman N., Yousif Z.M., Al-Falahi S. (2003). Aflatoxin M1 in breast-milk of UAE women. Ann. Trop. Paediatr..

[16] Anderson D.M., Williams F.H., Merkatz R.B., Schulman P.K., Kerr D.S., Pittard W.B. (1983). 3^rd^, Length of gestation and nutritional composition of human milk. Am. J. Clin. Nutr..

[17] Anderssen S.H., Løvlund E.E., Nygaard E.A., Selberg T.R., Størdal K. (2015). Expressing breast milk at home for 24-h periods provides viable samples for macronutrient analysis, Acta. Paediatr..

[18] Bauer J., Gerss J. (2011). Longitudinal analysis of macronutrients and minerals in human milk produced by mothers of preterm infants. Clin. Nutr..

[19] Beijers R.J., Graaf F.V., Schaafsma A., Siemensma A.D. (1992). Composition of premature breast-milk during lactation: constant digestible protein content (as in full term milk). Early Hum. Dev..

[20] Belfort M., Cherkerzian S., Bell K., Soldateli B., Cordova Ramos E., Palmer C. (2020). Macronutrient intake from human milk, infant growth, and body composition at term equivalent age: a longitudinal study of hospitalized very preterm infants. Nutrients.

[21] Bishara R., Dunn M.S., Merko S.E., Darling P. (2008). Nutrient composition of hindmilk produced by mothers of very low birth weight infants born at less than 28 weeks’ gestation. J. Hum. Lact.

[22] Brion L.P., Rosenfeld C.R., Heyne R., Brown L.S., Lair C.S., Petrosyan E. (2020). Optimizing individual nutrition in preterm very low birth weight infants: double-blinded randomized controlled trial. J. Perinatol.

[23] Britton J.R. (1986). Milk protein quality in mothers delivering prematurely: implications for infants in the intensive care unit nursery setting. J. Pediatr. Gastroenterol. Nutr..

[24] Bulut Ö., Çoban A., İnce Z. (2019). Macronutrient analysis of preterm human milk using mid-infrared spectrophotometry. J. Perinat. Med..

[25] Butte N.F., Garza C., Johnson C.A., Smith E.O., Nichols B.L. (1984). Longitudinal changes in milk composition of mothers delivering preterm and term infants. Early Hum. Dev..

[26] Caldeo V., Downey E., O'Shea C.A., Affolter M., Volger S., Courtet-Compondu M.C. (2021). Protein levels and protease activity in milk from mothers of pre-term infants: a prospective longitudinal study of human milk macronutrient composition. Clin. Nutr..

[27] Campbell-Yeo M.L., Allen A.C., Joseph K.S., Ledwidge J.M., Caddell K., Allen V.M. (2010). Effect of domperidone on the composition of preterm human breast milk. Pediatrics.

[28] Chuang C.K., Lin S.P., Lee H.C., Wang T.J., Shih Y.S., Huang F.Y. (2005). Free amino acids in full-term and pre-term human milk and infant formula. J. Pediatr. Gastroenterol. Nutr..

[29] Corvaglia L., Battistini B., Paoletti V., Aceti A., Capretti M.G., Faldella G. (2008). Near-infrared reflectance analysis to evaluate the nitrogen and fat content of human milk in neonatal intensive care units. Arch. Dis. Child Fetal Neonatal. Ed..

[30] Corvaglia L., Aceti A., Paoletti V., Mariani E., Patrono D., Ancora G. (2010). Standard fortification of preterm human milk fails to meet recommended protein intake: bedside evaluation by near-infrared-reflectance-analysis. Early Hum. Dev..

[31] Dingess K.A., de Waard M., Boeren S., Vervoort J., Lambers T.T., van Goudoever J.B. (2017). Human milk peptides differentiate between the preterm and term infant and across varying lactational stages. Food Funct.

[32] de Halleux V., Rigo J. (2013). Variability in human milk composition: benefit of individualized fortification in very-low-birth-weight infants. Am. J. Clin. Nutr..

[33] de Oliveira S.C., Bellanger A., Ménard O., Pladys P., Le Gouar Y., Dirson E. (2017). Impact of human milk pasteurization on gastric digestion in preterm infants: a randomized controlled trial. Am. J. Clin. Nutr..

[34] Ellis L., Picciano M.F., Smith A.M., Hamosh M., Mehta N.R. (1990). The impact of gestational length on human milk selenium concentration and glutathione peroxidase activity. Pediatr. Res..

[35] Elmlinger M.W., Hochhaus F., Loui A., Frommer K.W., Obladen M., Ranke M.B. (2007). Insulin-like growth factors and binding proteins in early milk from mothers of preterm and term infants. Horm. Res..

[36] Erickson T., Gill G., Chan G.M. (2013). The effects of acidification on human milk's cellular and nutritional content. J. Perinatol.

[37] Faerk J., Skafte L., Petersen S., Peitersen B., Michaelsen K.F. (2001). Macronutrients in milk from mothers delivering preterm. Adv. Exp. Med. Biol..

[38] Gates A., Marin T., De Leo G., Waller J.L., Stansfield B.K. (2021). Nutrient composition of preterm mother's milk and factors that influence nutrient content. Am. J. Clin. Nutr..

[39] Groh-Wargo S., Valentic J., Khaira S., Super D.M., Collin M. (2016). Human milk analysis using mid-infrared spectroscopy. Nutr. Clin. Pract..

[40] Gross S.J. (1983). Growth and biochemical response of preterm infants fed human milk or modified infant formula. N. Engl. J. Med..

[41] Gross S.J. (1987). Bone mineralization in preterm infants fed human milk with and without mineral supplementation. J. Pediatr..

[42] Hsu Y.C., Chen C.H., Lin M.C., Tsai C.R., Liang J.T., Wang T.M. (2014). Changes in preterm breast milk nutrient content in the first month. Pediatr. Neonatol..

[43] Kreissl A., Zwiauer V., Repa A., Binder C., Thanhaeuser M., Jilma B. (2016). Human milk analyser shows that the lactation period affects protein levels in preterm breastmilk. Acta Paediatr.

[44] Lemons J.A., Moye L., Hall D., Simmons M. (1982). Differences in the composition of preterm and term human milk during early lactation. Pediatr. Res..

[45] Lemons J.A., Reyman D., Moye L. (1983). Amino acid composition of preterm and term breast milk during early lactation. Early Hum. Dev..

[46] Lepage G., Collet S., Bouglé D., Kien L.C., Lepage D., Dallaire L. (1984). The composition of preterm milk in relation to the degree of prematurity. Am. J. Clin. Nutr..

[47] Lev H.M., Ovental A., Mandel D., Mimouni F.B., Marom R., Lubetzky R. (2014). Major losses of fat, carbohydrates and energy content of preterm human milk frozen at -80°C. J. Perinatol.

[48] Maas C., Franz A.R., Shunova A., Mathes M., Bleeker C., Poets C.F. (2017). Choline and polyunsaturated fatty acids in preterm infants' maternal milk. Eur. J. Nutr..

[49] Maas Y.G., Gerritsen J., Hart A.A., Hadders-Algra M., Ruijter J.M., Tamminga P. (1998). Development of macronutrient composition of very preterm human milk. Br. J. Nutr..

[50] Maly J., Burianova I., Vitkova V., Ticha E., Navratilova M., Cermakova E. (2019). Preterm human milk macronutrient concentration is independent of gestational age at birth. Arch. Dis. Child Fetal Neonatal..

[51] McLeod G., Sherriff J., Nathan E., Hartmann P.E., Simmer K. (2013). Four-week nutritional audit of preterm infants born <33 weeks gestation. J. Paediatr. Child Health.

[52] McLeod G., Simmer K., Sherriff J., Nathan E., Geddes D., Hartmann P. (2015). Feasibility study: assessing the influence of macronutrient intakes on preterm body composition, using air displacement plethysmography. J. Paediatr. Child Health.

[53] Minić S., Ješić M., Đurović D., Miletić S., Lugonja N., Marinković V. (2018). Redox properties of transitional milk from mothers of preterm infants. J. Paediatr. Child Health.

[54] Molinari C.E., Casadio Y.S., Hartmann B.T., Arthur P.G., Hartmann P.E. (2013). Longitudinal analysis of protein glycosylation and β-casein phosphorylation in term and preterm human milk during the first 2 months of lactation. Br. J. Nutr..

[55] Montagne P., Cuillière M.L., Molé C., Béné M.C., Faure G. (1999). Immunological and nutritional composition of human milk in relation to prematurity and mother's parity during the first 2 weeks of lactation. J. Pediatr. Gastroenterol. Nutr..

[56] Moran-Lev H., Mimouni F.B., Ovental A., Mangel L., Mandel D., Lubetzky R. (2015). Circadian macronutrients variations over the first 7 weeks of human milk feeding of preterm infants. Breastfeed Med.

[57] Morton J., Wong R.J., Hall J.Y., Pang W.W., Lai C.T., Lui J. (2012). Combining hand techniques with electric pumping increases the caloric content of milk in mothers of preterm infants. J. Perinatol.

[58] Nielsen S.D., Beverly R.L., Underwood M.A., Dallas D.C. (2020). Differences and similarities in the peptide profile of preterm and term mother's milk, and preterm and term infant gastric samples. Nutrients.

[59] Norrgrann M., Hörnfeldt M., Latheef F., Blomqvist Y.T., Larsson A., Paulsson M. (2023). Lipid peroxidation and antioxidative capacity are unaltered in transitional breast milk exposed to light from women giving birth to preterm infants before 32 weeks of gestation. Nutrients.

[60] Paulaviciene I.J., Liubsys A., Molyte A., Eidukaite A., Usonis V. (2020). Circadian changes in the composition of human milk macronutrients depending on pregnancy duration: a cross-sectional study. Int. Breastfeed J..

[61] Perrella S.L., Hepworth A.R., Simmer K.N., Geddes D.T. (2015). Influences of breast milk composition on gastric emptying in preterm infants. J. Pediatr. Gastroenterol. Nutr..

[62] Radmacher P.G., Lewis S.L., Adamkin D.H. (2013). Individualizing fortification of human milk using real time human milk analysis, J. Neonatal. Perinatal. Med.

[63] Saarela T., Kokkonen J., Koivisto M. (2005). Macronutrient and energy contents of human milk fractions during the first six months of lactation. Acta. Paediatr..

[64] Sahin S., Ozdemir T., Katipoglu N., Akcan A.B., Kaynak Turkmen M. (2020). Comparison of changes in breast milk macronutrient content during the first month in preterm and term infants. Breastfeed. Med..

[65] Sann L., Bienvenu F., Lahet C., Bienvenu J., Bethenod M. (1981). Comparison of the composition of breast milk from mothers of term and preterm infants, Acta. Paediatr. Scand..

[66] Sauer C.W., Boutin M.A., Kim J.H. (2017). Wide variability in caloric density of expressed human milk can lead to major underestimation or overestimation of nutrient content. J. Hum. Lact.

[67] Smilowitz J.T., Gho D.S., Mirmiran M., German J.B., Underwood M.A. (2014). Rapid measurement of human milk macronutrients in the neonatal intensive care unit: accuracy and precision of Fourier transform mid-infrared spectroscopy. J. Hum. Lact.

[68] Stein H., Cohen D., Herman A.A., Rissik J., Ellis U., Bolton K. (1986). Pooled pasteurized breast milk and untreated own mother's milk in the feeding of very low birth weight babies: a randomized controlled trial. J. Pediatr. Gastroenterol. Nutr..

[69] Stevens L.H. (1969). The first kilogram. The protein content of breast milk on mothers of babies of low birth weight. Med. J. Aust..

[70] Stoltz Sjöström E., Ohlund I., Tornevi A., Domellöf M. (2014). Intake and macronutrient content of human milk given to extremely preterm infants. J. Hum. Lact.

[71] Tanaka M., Date M., Miura K., Ito M., Mizuno N., Mizuno K. (2023). Protein and immune component content of donor human milk in Japan: variation with gestational and postpartum age. Nutrients.

[72] Thomas M.R., Chan G.M., Book L.S. (1986). Comparison of macronutrient concentration of preterm human milk between two milk expression techniques and two techniques for quantitation of energy. J. Pediatr. Gastroenterol. Nutr..

[73] Zachariassen G., Fenger-Gron J., Hviid M.V., Halken S. (2013). The content of macronutrients in milk from mothers of very preterm infants is highly variable, Dan. Med. J..

[74] de Oliveira S.C., Bourlieu C., Ménard O., Bellanger A., Henry G., Rousseau F. (2016). Impact of pasteurization of human milk on preterm newborn in vitro digestion: gastrointestinal disintegration, lipolysis and proteolysis. Food Chem.

[75] Pamblanco M., Portolés M., Paredes C., Ten A., Comín J. (1989). Free amino acids in preterm and term milk from mothers delivering appropriate- or small-for-gestational-age infants. Am. J. Clin. Nutr..

[76] Rivera Velez S.M., Newkirk M., Roux A., Ellis G., Harlan R., Go M.D.A. (2023). Characterization of D-amino acids in colostral, transitional, and mature preterm human milk. Amino Acids.

[77] Spevacek A.R., Smilowitz J.T., Chin E.L., Underwood M.A., German J.B., Slupsky C.M. (2015). Infant maturity at birth reveals minor differences in the maternal milk metabolome in the first month of lactation. J. Nutr..

[78] Roelants J.A., Vlaardingerbroek H., van den Akker C.H.P., de Jonge R.C.J., van Goudoever J.B., Vermeulen M.J. (2018). Two-year follow-up of a randomized controlled nutrition intervention trial in very low-birth-weight infants. JPEN J. Parenter. Enteral. Nutr..

[79] Rozé J.C., Morel B., Lapillonne A., Marret S., Guellec I., Darmaun D. (2021). Association between early amino acid intake and full-scale IQ at age 5 years among infants born at less than 30 weeks' gestation. JAMA Netw. Open.

[80] Lönnerdal B. (2003). Nutritional and physiologic significance of human milk proteins. Am. J. Clin. Nutr..

[81] Zhang Z., Adelman A.S., Rai D., Boettcher J., Lőnnerdal B. (2013). Amino acid profiles in term and preterm human milk through lactation: a systematic review. Nutrients.

[82] van Sadelhoff J.H.J., Wiertsema S.P., Garssen J., Hogenkamp A. (2020). Free amino acids in human milk: a potential role for glutamine and glutamate in the protection against neonatal allergies and infections. Front. Immunol..

[83] Giuffrida F., Austin S., Cuany D., Sanchez-Bridge B., Longet K., Bertschy E. (2019). Comparison of macronutrient content in human milk measured by mid-infrared human milk analyzer and reference methods. J. Perinatol.

[84] Bergqvist Y., Karlsson L., Fohlin L. (1989). Total protein determined in human breast milk by use of coomassie brilliant blue and centrifugal analysis. Clin. Chem..

[85] Donovan S.M., Lönnerdal B. (1989). Development of a human milk protein standard. Acta Paediatr. Scand..

[86] Wu X., Jackson R.T., Khan S.A., Ahuja J., Pehrsson P.R. (2018). Human milk nutrient composition in the United States: current knowledge, challenges, and research needs. Curr. Dev. Nutr..

[87] Vieira A.A., Soares F.V.M., Pimenta H.P., Abranches A.D., Moreira M.E.L. (2011). Analysis of the influence of pasteurization, freezing/thawing, and offer processes on human milk's macronutrient concentrations. Early Hum. Dev..

[88] Binder C., Baumgartner-Parzer S., Gard L.-I., Berger A., Thajer A. (2023). Human milk processing and its effect on protein and leptin concentrations. Nutrients.

[89] Perrin M.T., Belfort M.B., Hagadorn J.I., McGrath J.M., Taylor S.N., Tosi L.M. (2020). The nutritional composition and energy content of donor human milk: a systematic review. Adv. Nutr..

